# Impaired embryonic motility in *dusp27* mutants reveals a developmental defect in myofibril structure

**DOI:** 10.1242/dmm.013235

**Published:** 2013-11-07

**Authors:** Kandice Fero, Sadie A. Bergeron, Eric J. Horstick, Hiba Codore, Grace H. Li, Fumihito Ono, James J. Dowling, Harold A. Burgess

**Affiliations:** 1 Program in Genomics of Differentiation, Eunice Kennedy Shriver National Institute of Child Health and Human Development, Bethesda, MD 20892, USA.; 2 Department of Pediatrics, University of Michigan Medical Center, Ann Arbor, MI 48109, USA.; 3 Department of Neurology, University of Michigan Medical Center, Ann Arbor, MI 48109, USA.; 4 Department of Neuroscience, University of Michigan Medical Center, Ann Arbor, MI 48109, USA.; 5 Section on Model Synaptic Systems, Laboratory of Molecular Physiology, National Institute on Alcohol Abuse and Alcoholism, Rockville, MD 20852, USA.; 6 Division of Neurology, Hospital for Sick Children, Toronto, ON M5G 1X8, Canada.; 7 Program for Genetics and Genome Biology, Hospital for Sick Children, Toronto, ON M5G 1X8, Canada.; 8 Department of Paediatrics, University of Toronto, Toronto, ON M5G 1X8, Canada.; 9 Department of Molecular Genetics, University of Toronto, Toronto, ON M5G 1X8, Canada.

**Keywords:** Zebrafish, *dusp27*, Motility, Myofibrillogenesis, Muscle

## Abstract

An essential step in muscle fiber maturation is the assembly of highly ordered myofibrils that are required for contraction. Much remains unknown about the molecular mechanisms governing the formation of the contractile apparatus. We identified an early embryonic motility mutant in zebrafish caused by integration of a transgene into the pseudophosphatase *dual specificity phosphatase 27* (*dusp27*) gene. *dusp27* mutants exhibit near complete paralysis at embryonic and larval stages, producing extremely low levels of spontaneous coiling movements and a greatly diminished touch response. Loss of *dusp27* does not prevent somitogenesis but results in severe disorganization of the contractile apparatus in muscle fibers. Sarcomeric structures in mutants are almost entirely absent and only rare triads are observed. These findings are the first to implicate a functional role of *dusp27* as a gene required for myofiber maturation and provide an animal model for analyzing the mechanisms governing myofibril assembly.

## INTRODUCTION

Animal models of neuromuscular disorders are an invaluable resource for both identifying genes that are required for normal development of the neuromuscular system (Pappalardo et al., 2013) and elucidating the pathogenic mechanisms of human disease genes (reviewed by Berger and Currie, 2012). In zebrafish, precise genetic processes governing muscle and nerve development result in highly stereotyped early behaviors that are easily observed. The earliest motor behavior – internally generated spontaneous coiling movements – occurs within 18 hours post-fertilization (hpf) (Grunwald et al., 1988). By 21 hours, embryos show a vigorous tail contraction in response to touch (Saint-Amant and Drapeau, 1998) and a few hours later also respond to light (Kokel et al., 2010). Rapid neural and muscular maturation enable this fast development of behavior. The first muscle fiber contractions occur just minutes after primary motor neurons emerge in a given somite, and large trunk flexions occur while somitogenesis is still ongoing (Myers et al., 1986). The first mutagenesis screens in zebrafish identified genetic mutants affecting nerve and muscle development by the disruptions caused to embryonic motility (Felsenfeld et al., 1990; Westerfield et al., 1990). Subsequently, systematic genetic screens and characterization of spontaneous mutations in wild-type fish stocks led to the isolation of genes required for many aspects of early motility, including neuronal specification, axon pathfinding, neuromuscular-junction formation, muscle differentiation and myofiber contraction (Granato et al., 1996; Hirata et al., 2007).

The high efficiency of transposon-mediated transgenesis has facilitated high-throughput transgenesis efforts, including enhancer and gene-trapping screens (Kawakami et al., 2004). The random insertion of a transgene into or nearby a gene can disrupt expression sufficiently to cause a mutant phenotype (Sivasubbu et al., 2006; Nagayoshi et al., 2008). A key advantage of gene disruption by transgenes is that mutations can be easily mapped, avoiding the time-consuming linkage analysis required to identify mutations induced by chemical exposure or radiation. We recently performed an enhancer-trap screen aimed at generating neuronal-specific transgenic lines useful for manipulating discrete cohorts of neurons (Bergeron et al., 2012). Of the integrations that we have mapped so far, 17% (7/41) are in an exon or the first intron of a gene, making them potentially mutagenic. One instance is the enhancer-trap line *Et(REx2-SCP1:Gal4ff)y241* (*y241*), in which we noted a strong embryonic motility phenotype. Here we show that this line harbors a transgene insertion that disrupts expression of the *dual specificity phosphatase 27* (*dusp27*) gene. Mutants show severe disruption of muscle architecture, including almost complete disorganization of the contractile apparatus. The *y241* mutant is a new myopathy model that might help to elucidate the molecular mechanisms governing the formation of the contractile machinery within myofibers.

## RESULTS

**Fig. 1.**
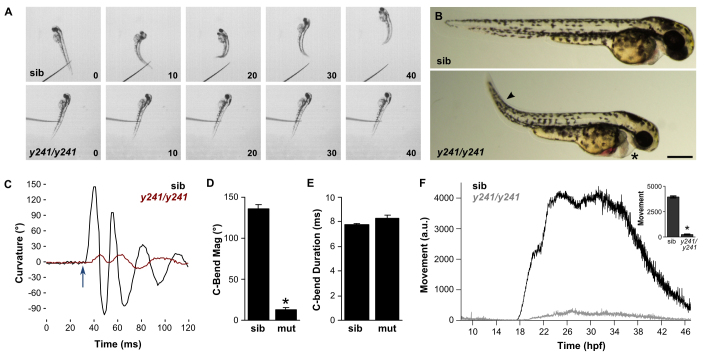
**Embryonic motility is impaired in *y241* mutant embryos.** (A) Swimming response to a tactile stimulus is severely impaired in 2 dpf *y241* mutants. Image frames are at 10 ms intervals from a touch stimulus delivered with a fine needle. Siblings (top panels) swim rapidly forward, whereas mutants (lower panels) respond with a barely perceptible shiver. (B) *y241* mutants (bottom panel) show edema adjacent to the heart (asterisk), tail curvature (arrowhead) and smaller eyes. Scale bar: 300 μm. (C) Curvature trace for 120 ms during an acoustic startle response in a sibling (black) and *y241* mutant (red) embryo. The stimulus was delivered at 30 ms (arrow). (D) The magnitude of the initial C-bend in *y241* mutant acoustic startle responses is reduced compared with that of wild-type embryos (*n*=6 larvae each), whereas (E) the duration of the initial C-bend is not significantly different. *P<0.001. (F) Spontaneous coiling is strongly reduced in *y241* mutants (gray, *n*=7) compared with wild-type siblings (black, *n*=37) during the entire period of early locomotor development. *y*-axis is arbitrary units. Inset, average coiling during 24–28 hpf for the same larvae. *P<0.001. Graphs show mean ± s.e.m.

During maintenance of the *y241* line, we observed embryos from multiple heterozygous incrosses that showed poor touch responsiveness. Consistent with the presence of a recessively segregating mutant allele, 24.4% of 2 days post-fertilization (dpf) embryos (154/630) responded to a tail touch with a weak ‘shiver’ instead of vigorous forward swimming (Fig. 1A). This phenotype was never observed in an outcross of *y241* transgenics to a wild-type strain of the same genetic background, confirming a recessive mode of inheritance (0/168 embryos). In addition, pectoral fin movement was almost completely absent. Morphologically, mutant embryos show edema surrounding the heart, curvature of the tail and reduced eye size (Fig. 1B). Heartbeat frequency was reduced in 2 dpf embryos [siblings: 167±3 beats per minute (bpm), mutants: 147±5 bpm, *n*=10 larvae; *t*_13.6_=3.2, *P*=0.006]. The tail curvature defect in mutants normalizes by 4 dpf. Edema also resolved in around half of the mutants such that larvae were morphologically normal, apart from a failure to inflate swim bladders. These larvae continued to show a reduced touch response and also failed to generate a normal acoustic startle response. The acoustic startle response is a rapid swimming movement in zebrafish that begins with a stereotyped flexion of the body to one side (C-bend). To quantify the movement defect, we analyzed kinematic parameters of C-bends of 5 dpf larvae to acoustic startle stimuli (Burgess and Granato, 2007). Larvae responded with a reduced body curvature (Fig. 1C,D) but normal C-bend duration (Fig. 1E). This suggests that the swimming defect is not due to uncoordinated timing of left-right muscle contractions, but reflects reduced muscle contraction. Because short-latency acoustic startle responses are all-or-nothing events independent of the intensity of the acoustic stimulus (Burgess and Granato, 2007), the reduction in motor response cannot be ascribed to attenuated sensory reception.

**RESOURCE IMPACT**

**Background**

Congenital myopathies are muscle disorders in which infants present with generalized weakness, usually at birth. No treatments are available for any congenital myopathy and genetic diagnosis is difficult because many of the disease genes for this group of disorders remain unknown. A more complete understanding of the molecular mechanisms underlying the different stages of myogenesis is crucial for the development of treatment and diagnostic options for congenital myopathy. A key stage in myofiber maturation is myofibrillogenesis. During this stage, actin, myosin and numerous accessory proteins are assembled into sarcomeres, the basic contractile units of muscles. Although many sarcomeric proteins have been identified, much less is known about the processes that enable them to assemble into regular arrays.

**Results**

In this study, the authors identify an embryonic motility mutant in zebrafish that displays weak muscle contractions from the very earliest stages of development. They demonstrate that the motility defect in the *y241* mutant is due to a transgenic insertion in *dual specificity phosphatase 27* (*dusp27*) that disrupts gene expression. The authors report that *dusp27*, which encodes a pseudophosphatase with no previously identified physiological function, is expressed in somites at early stages of development but that somitogenesis and patterning are normal in the mutant zebrafish. By contrast, histological and electron microscopy analysis of muscle tissue reveal a near complete defect in the assembly of the contractile apparatus in myofibers in the mutant.

**Implications and future directions**

The finding that *dusp27* is required for the formation of sarcomeres opens up a new line of investigation into the mechanisms governing myofibrillogenesis. Moreover, this study provides a new animal model with well characterized genetic, histological and behavioral defects that will enable investigations into the molecular pathways that control sarcomere assembly and that underlie the development of congenital myopathy.

To confirm that the defect in *y241* mutants was primarily a motor defect and not a sensory defect, we examined ‘spontaneous coiling’, a transient behavior starting at around 18 hpf in which embryos show vigorous tail movements that are generated by circuitry intrinsic to the spinal cord (Saint-Amant and Drapeau, 1998; Downes and Granato, 2006). We used automated video recording and image analysis to quantify spontaneous coiling (supplementary material Fig. S1) and found that this behavior in mutants was strongly reduced in magnitude from the earliest time points when compared with wild-type sibling embryos (Fig. 1F). We also observed that, despite a strong reduction in the magnitude of the tail coiling in mutants (supplementary material Movies 1, 2), the frequency of coiling events was normal (24 hpf, siblings: 0.087±0.006 Hz, mutants: 0.100±0.016 Hz, *n*=29 and 10 embryos, respectively, *t*_11.5_=0.75, *P*=0.46). This again suggests that neuronal control of the timing of contractions was not impaired. Because the timing of tail movements was normal but the magnitude of contractions was markedly reduced in both spontaneous coiling and acoustic startle responses, reduced motility in *y241* embryos is probably due to a primary defect in muscle or neuromuscular junctions leading to reduced contractile strength. Moreover, because there is a reduction in the earliest muscle contractions at the outset of spontaneous coiling, the defect is likely to represent a structural or functional abnormality rather than a progressive breakdown of muscle integrity.

**Fig. 2.**
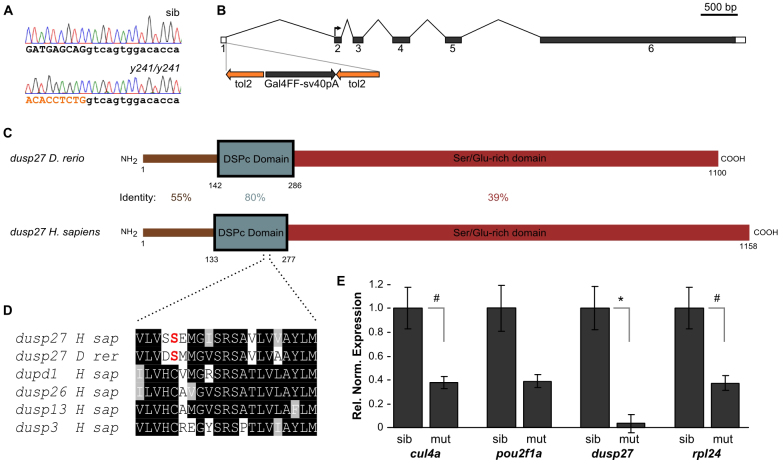
***dusp27* is disrupted in *y241* mutants.** (A) Chromatogram showing sequence of genomic DNA from *y241* wild-type siblings (top trace) and *y241* mutants (bottom trace). Black uppercase nucleotides are exon 1 of *dusp27*, lowercase bases are intron 1 and orange nucleotides are derived from the transgene. (B) Structure of the *dusp27* gene. Shaded areas represent predicted coding sequence with the translation start site in exon 2 marked. The transgene integration immediately following exon 1 in *y241* is represented, showing the orientation of the tol2 arms, Gal4FF and sv40 polyA signal. (C) The predicted proteins encoded by *dusp27* in zebrafish (NCBI XM_003197607) and human (NP_001073895) are 121.6 kDa and 130.2 kDa, respectively, and have highest identity in the DSPc phosphatase domain (boxed). The C-terminal (red) has relatively poor homology, but both are highly enriched in serine and glutamate residues (total 24.6% and 29.6% of all amino acids in zebrafish and human, respectively). (D) The catalytic cysteine in the core of the phosphatase domain is replaced by a serine (red) in *dusp27* in both human and zebrafish. For comparison, the four paralogs with the highest identity to human *DUSP27* are shown, each of which contains the catalytic cysteine residue. (E) Expression of genes (*cul4a*, *cullin4a; pou2f1a*, *POU class 2 homeobox 1a; rpl24*, *60S ribosomal protein L24*) in the immediate vicinity of the *y241* integration site as determined by RT-qPCR. Expression is calculated normalized to *ef1a*, relative to the sibling group for each gene. Comparison between sibling and mutant larvae from three independent clutches. ^#^*P*<0.05; *P<0.001. Graphs show mean ± s.e.m.

Linker-mediated PCR mapping determined that the transgene integration site in *y241* is on chromosome 1 at nucleotide 619473 (zebrafish genome Zv9; Fig. 2A). Genotyping showed that all mutant larvae (*n*=128) were homozygous for the transgenic allele, whereas unaffected siblings (*n*=41) were either wild-type or heterozygous for the transgenic allele, making it unlikely that an independently segregating mutation is responsible for the mutant phenotype. Measurement of swim distances in response to touch revealed no difference between wild-type embryos and embryos heterozygous for the transgene (*t*_29_=0.50, *P*=0.62).

Transcripts of the zebrafish homolog of *dusp27* map near this chromosomal location, but it was unclear whether the integration site fell within the gene or was in the upstream promoter region. We used 5′ RACE to determine the transcriptional start site of *dusp27* and found that the transgene insertion site is immediately after the last base of exon 1, thereby disrupting a near consensus 5′ splice donor site (Fig. 2A,B). Exon 1 contains a single ATG that is not in frame with the large open reading frame that begins near the start of exon 2. Dusp27 is a member of the dual-specificity phosphatase (DUSP) family, members of which share sequence similarity in the catalytic phosphatase domain. Zebrafish Dusp27 is most strongly similar (80% identity) to human DUSP27 in this domain, with weaker similarity in the N-terminal region and relatively poor identity (40%) in the extended C-terminal region, which, in both species, is markedly enriched in serine and glutamate residues (Fig. 2C). An unusual feature of Dusp27 is the loss of the highly conserved cysteine residue at the base of the catalytic loop in DUSP proteins, suggesting that Dusp27 is not an active phosphatase (Fig. 2D). Because the integration in *y241* likely disrupts splicing of *dusp27*, we measured transcript levels using quantitative RT-PCR (qPCR) for a region of *dusp27* encoded by exons 4–5 downstream of the integration site. *dusp27* expression is reduced to 3.6% of wild-type levels in mutant larvae (Fig. 2E), a finding confirmed by *in situ* hybridization for *dusp27* expression in mutants (supplementary material Fig. S2A), indicating that the transgene does indeed disrupt mRNA expression.

The transgene in *y241* includes two neuronal restrictive silencing elements (NRSEs) designed to suppress expression of the reporter outside the nervous system (Bergeron et al., 2012). NRSEs frequently occur at a distance from the genes they regulate, and act by binding the Rest protein, which recruits a protein complex to regulate chromatin structure (Huang et al., 1999; Ding et al., 2009). We therefore speculated that the transgene might also silence genes adjacent to *dusp27* and used qPCR to measure the expression level of the two adjacent genes (*rpl24* and *pou2f1a*) and a fourth gene, *cul4a*, with a large intron encompassing *dusp27*, *rpl24* and *pou2f1a*.

Expression of each of these genes was reduced to about 40% of the level in siblings, a reduction that was significant for *rpl24* and *cul4a* (Fig. 2E). To test whether the reduction in the expression of genes closest to the transgene integration site contributes to the mutant phenotype, we injected a morpholino against *rest* that we previously demonstrated can reverse transgene silencing in non-neuronal tissues (Bergeron et al., 2012) into *y241* homozygous embryos (Gates et al., 2010). Mutants injected with *rest* morpholino remained almost completely unresponsive to a touch stimulus, indistinguishable from mutants injected with control morpholino (2 dpf embryos: *n*=7 *y241* homozygotes injected with control morpholino, *n*=10 homozygotes injected with *rest* morpholino). This result affirms that the mutant phenotype is not caused by Rest-mediated silencing of genes proximal to the insertion site, but rather by disruption of *dusp27*.

**Fig. 3.**
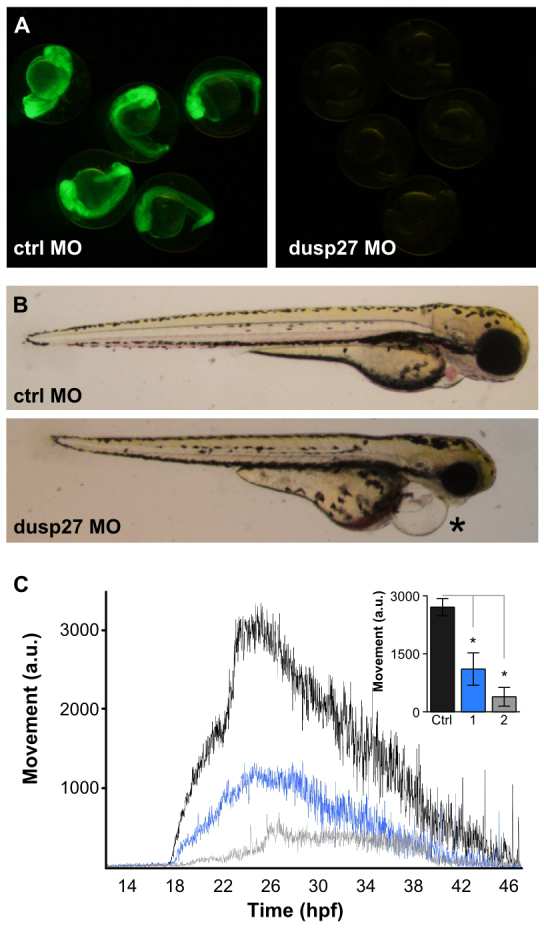
**Morpholino knockdown of *dusp27* phenocopies *y241* mutants.** (A) Validation of morpholino efficacy for *dusp27* knockdown using a *dusp27* N-terminal sequence fused to GFP. (B) Morphology of embryos injected with control morpholino (top panel) or 1 ng of morpholino against *dusp27* (bottom panel). Embryos show pronounced edema (asterisk). (C) Spontaneous coiling in embryos injected with control morpholino (black, *n*=11), 1 ng *dusp27* morpholino (blue, *n*=9) or 2 ng *dusp27* morpholino (gray, *n*=9). Inset, average coiling during 24–28 hpf for the same larvae. *P<0.01. Graphs show mean ± s.e.m.

To verify that loss of *dusp27* produces the mutant phenotype, we injected wild-type embryos with a translation-blocking morpholino against *dusp27* that was able to strongly suppress protein translation from an mRNA encoding a fusion of the morpholino target sequence to GFP (Fig. 3A). At 2 dpf, morphants were morphologically normal but showed pericardial edema and tail curvature (*n*=17/22 embryos; Fig. 3B) similar to phenotypes seen in the *y241* mutants. These phenotypes were not observed in embryos injected with control morpholino (*n*=32). Like *y241* mutants, *dusp27* morphants responded to a touch stimulus with only a weak shiver, whereas embryos injected with control morpholino executed a vigorous swim bout in response to touch. To quantify the movement defect in morphants, we measured spontaneous coiling. Morphants showed a dose-dependent reduction in movement throughout the entire period of spontaneous coiling, excluding the possibility that reduced movement reflected developmental delay (Fig. 3C; supplementary material Movies 3, 4). The close similarity of morphological and behavioral phenotypes caused by transgenic insertion into *dusp27* and injection of morpholino against *dusp27* demonstrates that loss of *dusp27* expression produces a profound locomotor impairment. This conclusion is supported by the rescue of the mutant histological phenotype by restoring expression of *dusp27* (see below).

**Fig. 4.**
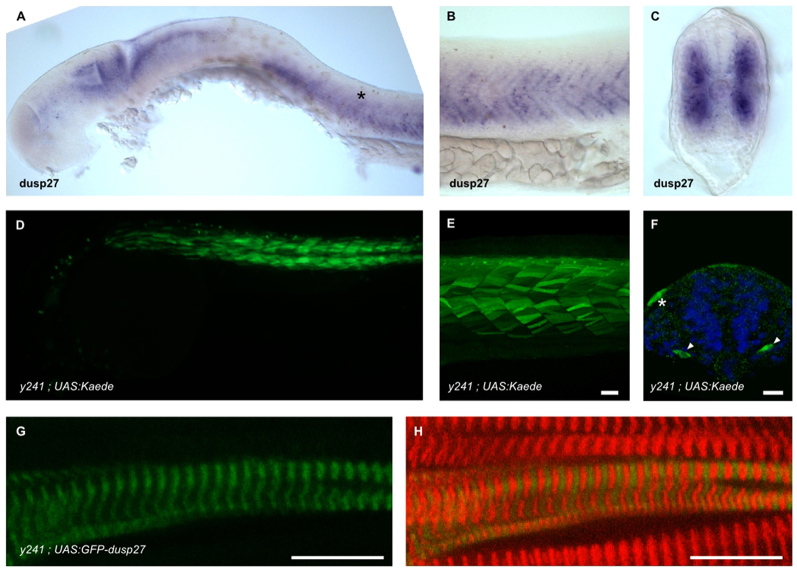
***dusp27* is expressed during embryonic muscle development.** (A–C) *In situ* hybridization for *dusp27* expression at 24 hpf. Prominent expression is seen in the somites (asterisk). (B) Lateral view above the yolk extension. (C) Cross-section through the trunk caudal to the yolk extension. (D–F) Kaede reporter expression in *y241; UAS:kaede* embryos. (D) Kaede expression is strongly expressed in trunk muscle at 24 hpf and (E) continues to label myofibers in 6 dpf larvae. Scale bar: 50 μm. (F) Early expression in somites is observed at 15 somites in a small number of cells adjacent to the notochord (arrowheads), as well as at the lateral edge of the somite (asterisk). Scale bar: 50 μm. (G,H) Expression of GFP-Dusp27 in muscle cells shows a striated pattern (green, G) that alternates with α-actinin (red in merge, H). Scale bars: 10 μm.

At 24 hpf, *dusp27* mRNA is expressed in the somites (Fig. 4A–C) and several regions of the brain, including the midbrain roof (tectum). Kaede expression in *y241* embryos recapitulates this pattern (Fig. 4D–F). Kaede is first detected in somites at 15 somites, is strongly expressed by 24 hpf and continues to be expressed in fast muscle fibers through 6 dpf. The earliest cells within the somites to express Kaede are found in medial positions near the notochord, and at the lateral edge of the somites (Fig. 4F). *dusp27* mRNA was expressed at 15 somites (supplementary material Fig. S2B) but too weakly to assess whether spatial organization was similar to the transgene. Within the brain, transgene expression is first seen in the optic tectum at 24 hpf, is robust by 48 hpf and continues through 6 dpf (supplementary material Fig. S2C). Expression of a GFP-Dusp27 fusion protein in muscle fibers revealed a regular striated pattern, indicating that Dusp27 might localize to sarcomeres (Fig. 4G,H). To determine whether abnormal neuronal activity contributes to the phenotype, we raised embryos from 6 hpf to 24 hpf in the sodium channel blocker tricaine (0.01%) to suppress electrical activity (Frazier and Narahashi, 1975). After washing out the tricaine, mutant embryos showed a very low level of spontaneous movement that was indistinguishable from mutants raised without tricaine (coiling activity in mutants raised in embryo medium only: 367±60 a.u.; with tricaine 384±33 a.u.; *t*_35.1_=−0.23, *P*=0.82), indicating that the motor impairment is not a developmental consequence of aberrant neuronal activity.

**Fig. 5.**
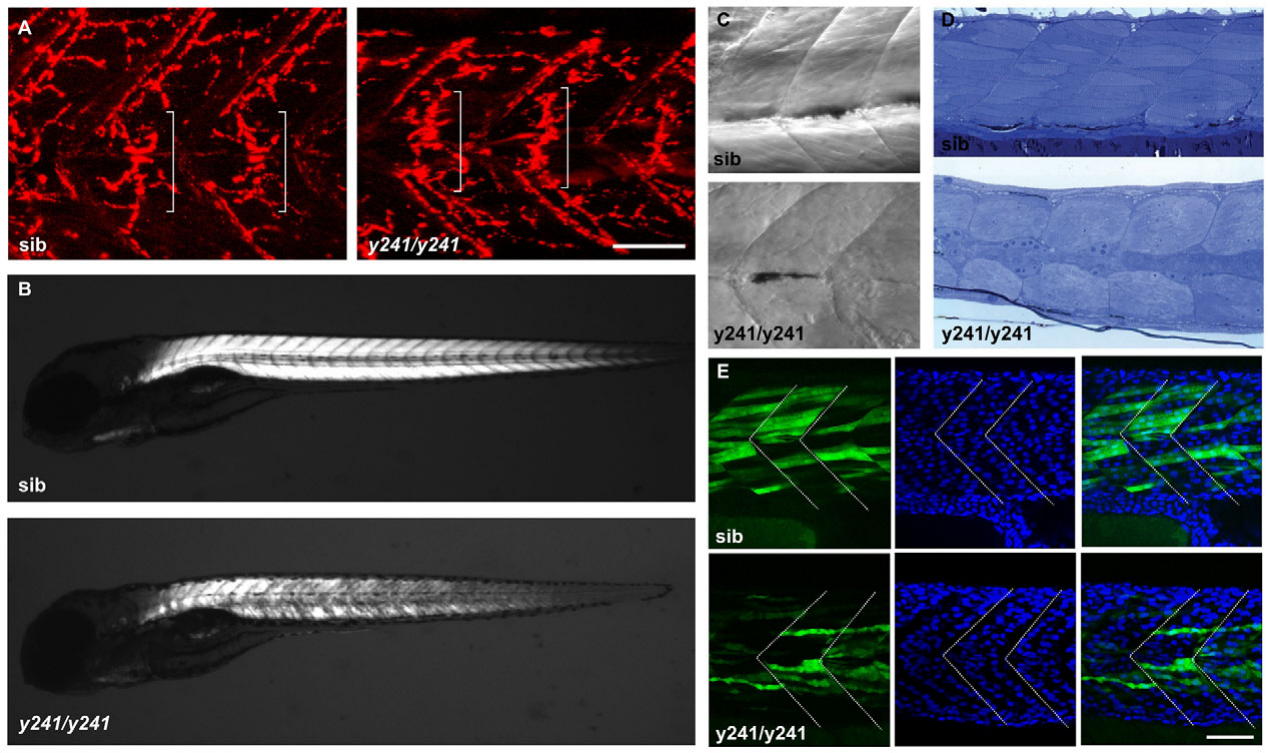
**Muscle integrity is impaired in *y241* mutants.** (A) α-bungarotoxin-conjugated Alexa-Fluor-555 staining of 2 dpf wild-type sibling (left) and *y241* mutant (right) embryos, showing equatorially located neuromuscular junctions (brackets). Scale bar: 50 μm. (B) Birefringence in live 5 dpf wild-type sibling (top) and *y241* mutant (bottom) larvae detected using polarized light. Mutants show reduced and patchy birefringence. (C) Dodt-gradient-contrast illumination of muscle in 3 dpf larvae after removal of skin. Internal muscle striations are visible in myofibers stretching between somite boundaries in siblings (top) but not in *y241* mutants (bottom). (D) 10 μm toluidine-blue-stained sections through the tail of 3 dpf wild-type siblings (top) and *y241* mutants (bottom). (E) Kaede expression in highly variegated 24 hpf *y241; UAS:kaede* sibling embryos (top panels) and mutants (bottom panels) with DAPI staining to reveal nuclear positions. Somite boundaries observed in bright-field images are indicated by dashed lines. Scale bar: 30 μm.

**Fig. 6.**
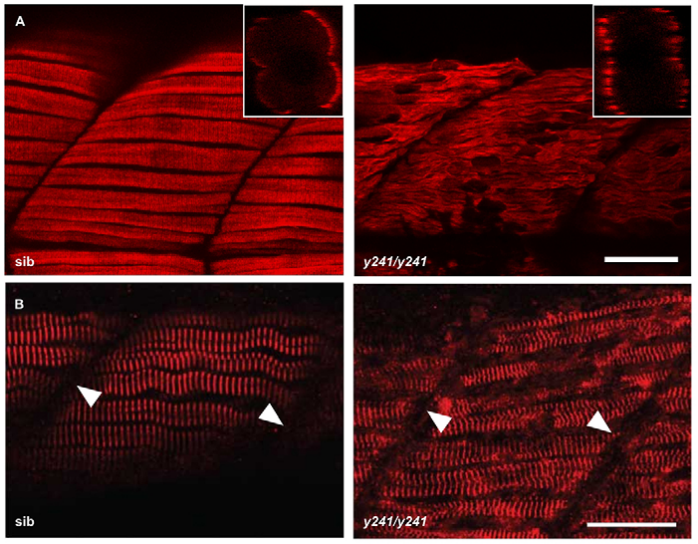
**Slow muscle myofibrils are partially disrupted in *y241* mutants.** (A) F59 staining against slow-twitch muscle myosin heavy chain in 48 hpf embryos shows the presence of slow muscle fibers at the lateral edge of the somite in both mutants and siblings but disorganized thick filaments in *y241* mutants. Scale bar: 20 μm. Insets show rotated transverse view through the somite. (B) α-actinin staining in slow-twitch fibers shows preserved Z-lines (arrowheads) in *y241* mutants. Scale bar: 25 μm.

The near normal timing but greatly reduced amplitude of muscle contractions suggests that *y241* mutants have a primary defect in the neuromuscular junction or muscle. Because α-bungarotoxin staining of neuromuscular junctions in mutant larvae failed to reveal an impairment (Fig. 5A) and *dusp27* is strongly expressed in muscle, we examined muscle structure. The characteristic chevron-shaped morphology of the somite boundaries was not disrupted and somite width was close to normal in mutants (at 4 dpf wild types: 82.4±0.9 μm; mutants 78.8±0.4 μm), indicating that somitogenesis is not disrupted in mutants. Supporting this, whole-mount *in situ* hybridization with a *myoD* probe on ten-somite-stage offspring (*n*=40) from *y241* heterozygote parents did not reveal embryos with altered expression that would indicate abnormal somite patterning (supplementary material Fig. S3A). In addition, localization of dystrophin demonstrated that somite boundaries are intact (supplementary material Fig. S3B). Muscle integrity in zebrafish is readily assessed by examining birefringence, an optical effect caused by the diffraction of polarized light through myofilaments (Granato et al., 1996). Birefringence was abnormal in *y241* mutants, suggesting a disorganization of muscle fibers (Fig. 5B). Indeed, muscle striation was lost in mutants (Fig. 5C) and muscle fibers appeared disorganized in toluidine-blue-stained semi-thin sections through the tail (Fig. 5D). In zebrafish, transgenic reporters controlled by UAS-element-containing promoters are susceptible to stochastic silencing, leading to mosaic reporter expression (Akitake et al., 2011). To determine whether the observed muscle defects were structural or an indirect result of the loss of attached muscle fibers, we took advantage of mosaic expression of the *UAS:kaede* transgene to visualize individual fast muscle fibers. Fast muscle fibers were multinucleated (Fig. 5E; supplementary material Fig. S4) and extended between somite boundaries in mutants, suggesting that muscle cells fuse and extend normally (Fig. 5E). Slow-twitch muscles were correctly located at the superficial aspect of the somite as revealed by F59 staining (Devoto et al., 1996) (Fig. 6A), and immunofluorescence against engrailed protein revealed normal localization of muscle pioneers at the horizontal myoseptum (supplementary material Fig. S3C) (Devoto et al., 1996). These findings suggested that muscle cells are present and correctly localized but internally disorganized in *y241* mutants, which led us to examine the subcellular architecture of myofibers.

**Fig. 7.**
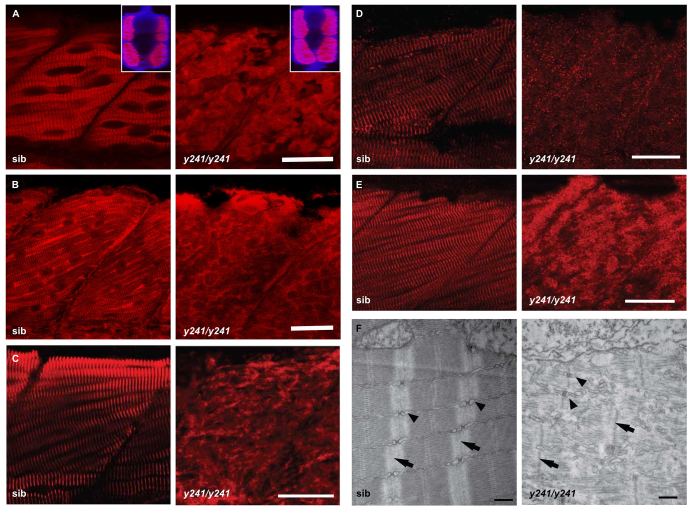
**Fast muscle myofibrils are disrupted in *y241* mutants.** (A–E) Immunofluorescence in fast-twitch fibers in 48 hpf sibling and *y241* mutant embryos. (A) F310 labeling of fast-twitch-fiber myosin shows disruption of thick filaments. Scale bar: 25 μm. Insets show rotated transverse view through the somite (merged view of DAPI in blue and F310 in red). (B) CH1 labeling of tropomyosin shows disruption of thin filaments. Scale bar: 20 μm. (C) α-actinin staining shows loss of Z-lines in fast-twitch fibers. Scale bar: 25 μm. (D) Anti-titin staining. Scale bar: 30 μm. (E) DHPR in fast-twitch fibers. Scale bar: 25 μm. (F) Transmission electron microscopy in myofibers of 3 dpf *y241* siblings and mutants, showing Z-lines (arrows) and triads (arrowheads). Scale bars: 500 nm.

Slow-twitch muscle myosin heavy chain showed a regular striated pattern in sibling larvae that was not present in *y241* mutants, indicating abnormal thick-filament assembly in slow-twitch fiber sarcomeres (Fig. 6A). In contrast, α-actinin staining showed regular striation in mutant slow-twitch fibers, demonstrating that Z-lines were present (Fig. 6B). The selective maintenance of Z-lines despite disorganization of other sarcomere components due to genetic mutation or pharmacological treatment has been previously observed (Funatsu et al., 1990; Li et al., 2009). In fast muscle fibers, staining against myosin light chain using the F310 antibody (Zeller et al., 2002) and tropomyosin with CH1 (Lin et al., 1985) demonstrated severe disruption of thick and thin filaments, respectively (Fig. 7A,B). Unlike in slow muscle fibers, Z-lines were also disrupted in fast-twitch fibers (Fig. 7C; also apparent in isolated fast-twitch myofibers, supplementary material Fig. S4). Mosaic expression of the *GFP-dusp27* transgene in *y241* homozygotes selectively rescued the striated pattern of α-actinin staining, further confirming that loss of *dusp27* is the basis of the structural phenotype (supplementary material Fig. S3D). The giant sarcomeric protein titin and proteins involved in excitation coupling – ryanodine receptors and dihydropyridine receptors – also failed to show striated expression within fast muscle fibers (Fig. 7D,E; supplementary material Fig. S3E). Expression of these muscle markers confirms that differentiated muscle cells are present in mutant embryos. However, these data suggest a near complete disorganization of the sarcomere and triad in fast muscle fibers. To verify this, we performed electron microscopy. Muscle tissue from *y241* mutants revealed massive disruption of the internal organization of myofibers (Fig. 7F). Although occasional triadic structures were visible, the ultrastructure of the t-tubules, sarcoplasmic reticulum and sarcomere were all highly disorganized in mutant embryos. These findings show that myofibrils are strongly disrupted in larvae lacking *dusp27* function. Because somitogenesis appears normal, but motility is lost from the earliest stages, our findings suggest that *dusp27* mutants have a primary defect in myofibrillogenesis.

## DISCUSSION

Here we show the first functional characterization associated with the loss of *dusp27*. Genetic or morpholino-mediated reduction of *dusp27* leads to a strong reduction in embryonic motility in zebrafish embryos due to massive disruption of myofibrils. Grossly normal somite morphology and patterning in mutants suggests that the defect is not in the specification of muscle precursors. Because the very first motor responses of the embryo already exhibit deficiencies and almost every aspect of myofiber internal organization is affected, it is likely that the mutation disrupts myofibril assembly rather than resulting in a progressive breakdown in muscle architecture (as might be observed in a dystrophy). Microarray analysis of genes activated during IGF-1-induced differentiation of cultured mouse myoblasts supports this theory – DUSP27 (referred to as hypothetical protein C130085G02) was found to be upregulated 4.6-fold within 24 hours of IGF-1 treatment (Kuninger et al., 2004).

Inconsistent nomenclature has been used to refer to *dusp27*. The closest human homolog to the zebrafish gene disrupted in *y241* (61% identity over 310 amino acids) is currently designated by the HUGO Gene Nomenclature committee as dual specificity phosphatase 27 (*DUSP27*, HUGO #25034, GenBank NM_001080426). Human *DUSP27* is on chromosome 1q24 and, like its zebrafish ortholog, is adjacent to a gene encoding POU class 2 homeobox 1. Zebrafish *dusp27* has a weaker match (43% identity over 169 amino acids) to the human gene dual specificity phosphatase and pro isomerase domain containing 1 (*DUPD1*, HUGO #23481, GenBank NM_001003892 on chr. 10q22). *DUPD1* has been referred to by several groups as *DUSP27* (Friedberg et al., 2007; Devi et al., 2011; Lountos et al., 2011), including in a recent review of DUSPs (Patterson et al., 2009). The only reports we are aware of which deal with *DUSP27* are GWAS and microarray studies (Kuninger et al., 2004; Iio et al., 2010; Nielsen et al., 2010).

Dusp27 contains a sequence that has strong similarity to the catalytic domain of DUSPs (DSPc domain). DUSPs play a key role in regulating intracellular signaling pathways in a variety of physiological contexts by hydrolyzing phosphorylated tyrosine, serine and threonine residues (Patterson et al., 2009). Dusp27 belongs to the ‘atypical’ subclass of DUSPs: this is a miscellaneous group, members of which are generally small (less than 30 kDa) and lack an N-terminal CH2 domain but are not close phylogenetic relatives (Patterson et al., 2009). *dusp27* is unusual among the atypical DUSPs in that it is large (130.2 kDa) and lacks the critical cysteine residue at the base of the catalytic cleft required for phosphatase activity. Despite its large size, outside of the DSPc domain, there are no recognizable protein domains in *dusp27*, making it difficult to predict its mode of action.

The best-studied role for DUSPs is in negative-feedback regulation of mitogen-activated kinase (MAPK) signaling cascades (reviewed by Lawan et al., 2012). MAPK phosphatases (MKPs) simultaneously dephosphorylate threonine and tyrosine residues within the MAPK activation loop to attenuate signaling in a wide variety of physiological contexts, including immune function, embryonic development and metabolic homeostasis. Recent studies have demonstrated roles for MKPs during development in zebrafish: *dusp4* is required for endoderm specification in zebrafish (Brown et al., 2008), and *dusp6* is required for embryonic axis formation and specification of cardiac progenitor cells regulating heart size (Tsang et al., 2004; Molina et al., 2009). MAPK has a well-established role in vertebrate somitogenesis. FGF signaling via MAPK regulates cell motility gradients in pre-somitic mesoderm that establish the rate of axis elongation and somite size (Sawada et al., 2001; Delfini et al., 2005; Bénazéraf et al., 2010). During somitogenesis, MAPK activity is high in posterior pre-somitic mesoderm and downregulated during maturation of anterior somites (Sawada et al., 2001). We speculated that defects in *y241* mutants might be due to a failure to attenuate MAPK signaling. However, raising embryos in U0126, which strongly inhibits ERK1/2 in zebrafish (Hawkins et al., 2008), did not restore motor function in mutants and no elevation in doubly phosphorylated ERK1/2 immunostaining was observed in mutants (data not shown), suggesting that the defect is not due to hyperactivity in the MAPK pathway.

It is likely that Dusp27 does not function as an MKP. Two residues in the catalytic core of all active DUSPs are not conserved in human and zebrafish Dusp27. In Dusp27, the nucleophilic cysteine is replaced by a serine residue. The same mutation introduced into Dusp6 produces a dominant-negative protein (Tsang et al., 2004) and abolishes enzymatic activity in several other DUSPs (Zhou et al., 1994; Aoki et al., 2001). Biochemical characterization is essential to confirm the loss of enzymatic activity; however, it is likely that Dusp27 is a pseudophosphatase. Although catalytically inactive, pseudoenzymes frequently have essential roles, regulating cell function through protein interactions (Leslie, 2013). Like Dusp27, the DUSP-like pseudophosphatase STYXL1 has a serine in place of the catalytic cysteine but is nevertheless required for sperm production in mice (Wishart and Dixon, 2002). Catalytically inactive DUSPs might act by binding to and regulating the activity of related active DUSPs, competing with active DUSPs for phospho-protein targets or by regulating the subcellular localization of binding partners. The latter possibility is suggested by the finding that type II DUSPs, characterized by nuclear export sequences, can sequester MAPK in the cytoplasm and prevent phosphorylation of nuclear targets (Brunet et al., 1999).

Zebrafish models of neuromuscular disorders have helped to clarify the pathogenic mechanisms of human disease genes (e.g. Telfer et al., 2012; Horstick et al., 2013; Mitsuhashi et al., 2013). The genetic basis of numerous subtypes of congenital myopathies are in genes that provide structural integrity of the muscle contractile apparatus, or calcium homeostasis within myofibers (Nance et al., 2012). The availability of animal models with comparable genetic mutations has helped to identify molecular pathways associated with disease pathogenesis and development of therapeutic strategies. This work is the first to reveal a role for the pseudophosphatase *dusp27* in the maturation of the contractile apparatus in myofibers. The molecular pathways through which it acts are at present unknown but the near complete lack of movement from the earliest stages of embryonic motility together with the extensive disorganization of muscle fiber architecture argue for an early and essential role in myofibrillogenesis.

## MATERIALS AND METHODS

### Zebrafish husbandry

All zebrafish in this study were maintained on a Tubingen long-fin strain background. Transgenic *Et(REx2-SCP1:Gal4ff)y241* fish were generated during an enhancer-trap screen in which the reduced-toxicity Gal4 variant Gal4FF (Asakawa et al., 2008) is downstream of a basal super core 1 *(SCP1)* promoter (Juven-Gershon et al., 2006) fused to tandem NRSEs (Bergeron et al., 2012). The NRSEs bind the transcriptional silencer Rest, which is expressed outside the nervous system, resulting in neural-specific expression in 30% of lines obtained with the *REx2-SCP1* promoter (Bergeron et al., 2012). *y241* is a case in which robust non-neuronal expression is observed. Gal4 expression was visualized with Kaede reporter line *Tg(UAS:Kaede)s1999t* (Davison et al., 2007). *y241* was maintained as a heterozygous stock because homozygotes are non-viable, dying by 11 dpf presumably owing to inability to feed. All *in vivo* experimental protocols were approved by the local Animal Care and Use Committee.

### *In situ* hybridization and immunohistochemistry

Larvae were fixed in 4% paraformaldehyde. Standard colorimetric whole-mount *in situ* hybridization (WISH) and immunohistochemistry was performed as described (Bergeron et al., 2008). For WISH, probes were *myoD* (Weinberg et al., 1996) and a *dusp27* fragment amplified by RT-PCR from 24 hpf embryonic RNA (primers 5′-TCTCTCAGAGCGACAAGACG-3′ and 5′-CAGGTAAGCAGCTACGAGCA-3′) and cloned into pGEM-T Easy (Promega, Madison, WI). No staining was observed with the sense probe. Antibodies used for immunofluorescence were anti-dystrophin [1:200; MANDRA1 (7A10) Developmental Studies Hybridoma Bank (DSHB), Iowa City, IA], anti-RYR (1:200; 34C DSHB), anti-DHPR α-subunit (1:200; 1A, Affinity Bioreagents, Golden, CO), anti-α-actinin (1:200; A7811 Sigma, St Louis, MO), anti-engrailed (1:500; 4D9, DSHB), F59 (1:500; F59, DSHB), F310 (1:500; DSHB), CH1 (1:500; DSHB), antititin (1:500; T11, Sigma), anti-kaede (1:200; PM012, MBL International, Woburn, MA), anti-mouse IgG Alexa Fluor 488 and 555 (1:800 and 1:1000, respectively; Invitrogen, Carlsbad, CA).

### Histology

For transmission electron microscopy, samples were fixed in 2.5% glutaraldehyde in 0.1 M Sorensen’s buffer, pH 7.4, overnight at 4°C. After several buffer rinses, they were post-fixed in 1% osmium tetroxide in the same buffer. Samples were rinsed in double distilled water to remove phosphate salt and then en bloc stained with aqueous 3% uranyl acetate for 1 hour. The preparation was dehydrated in ascending concentrations of ethanol, rinsed two times in propylene oxide and embedded in epoxy resin. The samples were ultra-thin sectioned 70 nm in thickness and stained with uranyl acetate and lead citrate. The sections were examined using a Philips CM100 electron microscope at 60 kV. Images were recorded digitally using a Hamamatsu ORCA-HR digital camera system operated using AMT software (Advanced Microscopy Techniques Corp., Danvers, MA). Semithin section preparations were made by cutting 0.5 μm sections through epon-embedded embryos and subsequently staining with toluidine blue. Sections were imaged on an Olympus BX43 microscope. Birefringence was measured on 5 dpf embryos anesthetized with tricaine. Images were taken using two polarizing filters on a Nikon AZ100 microscope; exposure was set at 100 μs for all images. α-bungarotoxin-conjugated Alexa-Fluor-555 staining for acetylcholine receptors at neuromuscular junctions was performed as previously described (Ono et al., 2001).

### Behavioral analysis

Larval startle responses in 5 dpf embryos were elicited using a minishaker (4810, Brüel and Kjaer, Norcross, GA) and tracked as previously described (Burgess and Granato, 2007) with the modification that kinematics for mutant larvae were manually extracted from curvature traces because responses were of too small magnitude to be automatically detected by Flote. Spontaneous coiling was measured in embryos arrayed in a 7×7 grid and illuminated with infrared light (Advanced Illumination, Rochester, VT) while imaged at 1 Hz (μEye IDS-1545LE-M, 1stVision, Andover, MA). Analysis was performed by adding a new tracking module in the DAQtimer software (Yokogawa et al., 2012) implementing a similar algorithm to that described previously (Myers et al., 1997). Briefly, pixels with an intensity change above a predetermined threshold between frames were counted and averaged each minute to provide a measure of overall movement, similar to counting ‘beam breaks’ to assess locomotor activity (supplementary material Fig. S1). Note that this method provides an aggregate measure of activity, combining frequency of tail coiling with the magnitude of tail movements, and the temporal profile of activity therefore differs slightly from previous analyses (Myers et al., 1997; Saint-Amant and Drapeau, 1998). Supplementary material Movies 1 and 2 were taken using a Leica stereomicroscope and an Olympus DP72 camera.

### Transgene mapping

*y241* was mapped using linker-mediated PCR with *Dpn*II linkers (Dupuy et al., 2005; Davison et al., 2007). Genotyping primers are x210g 5′-GTCCTCAGAGCTGGAGATCG-3′, x210r 5′-CCCTGATCAGTGTGTGGTGT-3′ and x210t 5′-TCAAGTAAAGTAAAAATCCCCAAAA-3′. The wild-type band is 241 bp and transgenic band 121 bp. Sequencing of the bands confirmed that they are derived from the wild-type locus and the junction of the transgene and wild-type genomic DNA, respectively.

### RT-qPCR

Total RNA was extracted from 3 dpf larvae (5–10 pooled larvae per treatment group), using Trizol reagent (Invitrogen) following the manufacturer’s protocol. cDNA was synthesized from RNA samples using iScript Advanced cDNA Synthesis Kit (Bio-Rad, Hercules, CA) using the oligo(dT) protocol. *dusp27* and neighboring genes *cul4a* (Ensembl ID ENSDART00000126069), *rpl24* (ENSDART00000019227) and *pouf1a* (ENSDART00000064142) were targeted for RT-qPCR using the following primers: *dusp27,* 5′-CATCGCTGAGAAGTCAGTGG-3′ and 5′-GGATGTTCATGCCGGAGTAG-3′ (spans exons 4–5); *pouf1a,* 5′-GTTTGAACCCCACCATCATC-3′ and 5′-GAGCCTGAATAGTGGCCAGA-3′ (spans exons 3–5); *cul4a,* 5′-CTGCTGAGATGGTGAAGCTG-3′ and 5′-CTGTCTTCAGCACTGCGTGT-3′ (spans exons 5–6); *rpl24,* 5′-TGAGGAGGTGTCGAAGAAGC-3′ and 5′-GCACTTCAGGCTTCTGGTTC-3′ (spans exons 3–4). The reference gene was *ef1a,* 5′-CTGGAGGCCAGCTCAAACAT-3′ and 5′-ATCAAGAAGAGTAGTACCGCTAGCAT-3′ (Tang et al., 2007). RT-qPCR was performed using a CFX96 C1000 Touch Thermal Cycler (Bio-Rad) and SsoAdvanced SYBR Green Supermix (Bio-Rad). For all genes, technical triplicates were used for each of the three biological replicates (different clutches of embryos). From standard curves, all target primer efficiencies were between 90% and 110%; standard curve *r*^2^ values were greater than 0.98, indicating low replicate variability. Expression data was then analyzed with CFX manager 3.0 software (Bio-Rad) using the ∆∆Cq method to determine relative normalized expression of target genes (Livak and Schmittgen, 2001).

### Morpholino injection

The translation-blocking morpholino against *dusp27* targets the region surrounding the start codon (5′-GATCCTCCACAGACGACGCCATCA-3′; Gene Tools, Philomath, OR). To confirm efficacy, we co-injected 2 ng of *dusp27* or control morpholino with 50 pg *in vitro* transcribed mRNA containing the morpholino target sequence fused to the 5′ of GFP.

### Rescue

To make the *UAS:GFP-dusp27* rescue construct, we synthesized zebrafish codon optimized emerald GFP (Genscript) and subcloned it into pT1UMP, removing the stop codon (Yokogawa et al., 2012). Zebrafish *dusp27* (ZGC *dusp27* cDNA clone 7420637, Thermo Scientific) was cloned in frame after the C-terminus of GFP. Embryos were injected with 75 pg of the rescue construct.

### Statistical analysis

SPSS (IBM, Armonk, NY) and Gnumeric (https://projects.gnome.org/gnumeric/) were used for statistical analysis. Statistical analysis was performed using Student’s *t*-test with a significance threshold of 0.05. Variation is presented as mean ± s.e.m. throughout the text.

## Supplementary Material

Supplementary Material
