# Supplementary material for: Impaired embryonic motility in dusp27 mutants reveals a developmental defect in myofibril structure
Source: Dis Model Mech. 2013 Nov 7;7(2):289–98. doi: 10.1242/dmm.013235 (PMC3917250; doi:10.1242/dmm.013235)
Supplement: Supplementary Material [file supp_7_2_289__index.html]

Impaired embryonic motility in dusp27 mutants reveals a developmental defect in myofibril structure — Supplementary Material 

# Impaired embryonic motility in *dusp27* mutants reveals a developmental defect in myofibril structure

## DMM013235 Supplementary Material

**Files in this Data Supplement:**

- **Supplementary Material PDF**
