# Supplementary material for: Impaired embryonic motility in dusp27 mutants reveals a developmental defect in myofibril structure
Source: Dis Model Mech. 2013 Nov 7;7(2):289–98. doi: 10.1242/dmm.013235 (PMC3917250; doi:10.1242/dmm.013235)
Supplement: Supplementary Material [file supp_7.2.289_DMM013235.pdf]

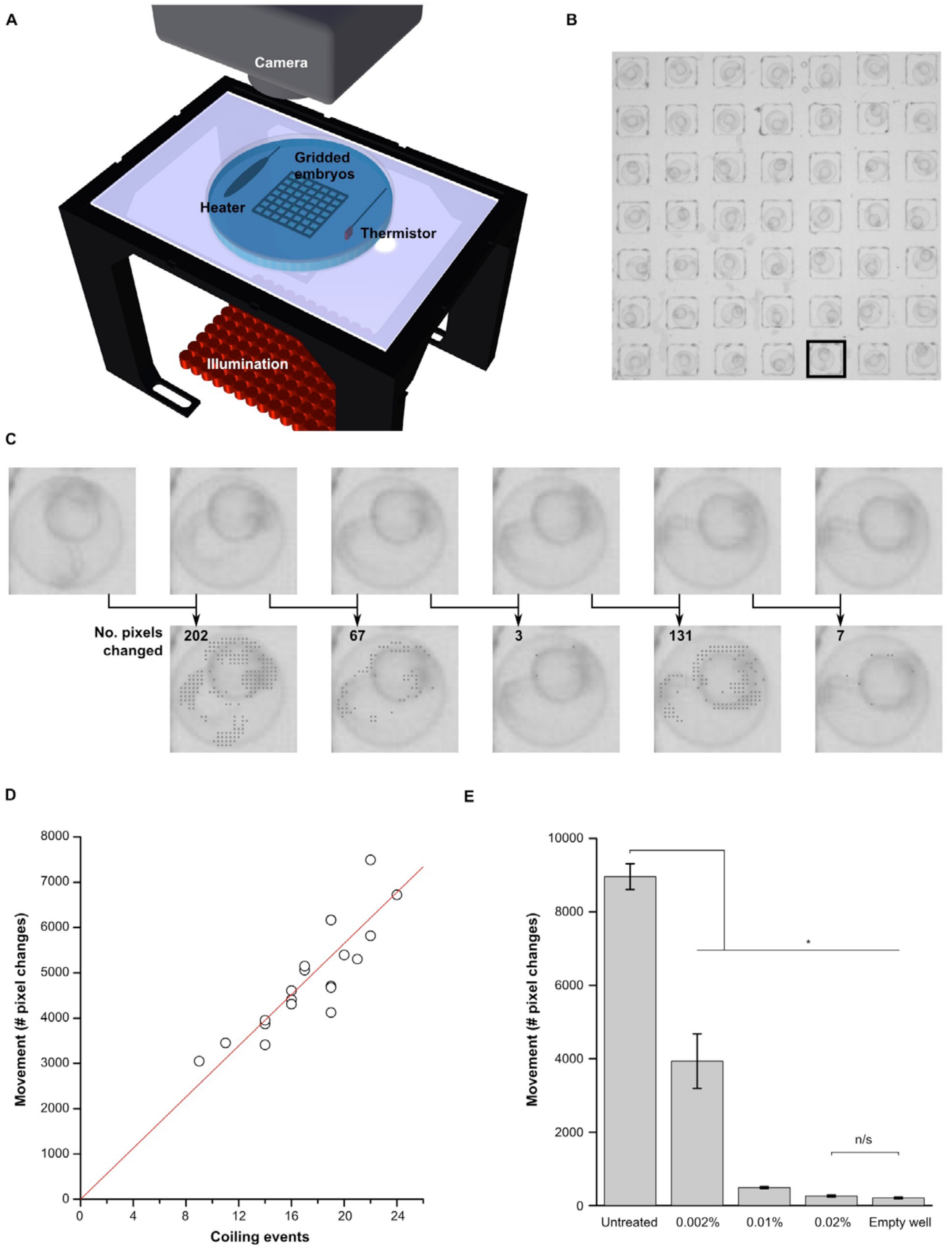

Fig. S1. Measurement of embryonic motor activity. (A) Schematic of the apparatus used to record motor activity in embryos. Embryos are arrayed in a 49 well grid in a Petri dish with temperature maintained at 28°C using a thermistor and heating element. A camera mounted above records images (B) at a 1 Hz framerate. (C) Motor activity is measured as the sum of the number of pixels which change in intensity greater or less than a predetermined threshold between frames. Top row shows a sequence of frames for the boxed well in (B), while the bottom row shows the same frames, with changed pixels highlighted and the total number of changed pixels indicated. To allow real-time processing, only every second pixel in the image is checked. (D) For validation, the coiling frequency was manually observed for 20 embryos (number of coiling events in a one minute interval) and the total number of pixel change events automatically measured for the same video sequence. Manual and automatically scored values are highly correlated Pearson r=0.86, $P<0.001$ confirming that the algorithm captures differences in motor activity. (E) For additional validation, embryos were exposed to varying concentrations of tricaine for 20 minutes ($N=14$ each condition) before movement was measured over a 10 minute period. The y-axis shows the mean number of pixel changes per minute averaged over the recording period. 0.002% tricaine significantly reduced but did not eliminate movement while 0.02% tricaine completely paralyzed embryos such that the number of pixel changes was not significantly (n/s) different from baseline as measured in wells with no embryos. * $P<0.001$.

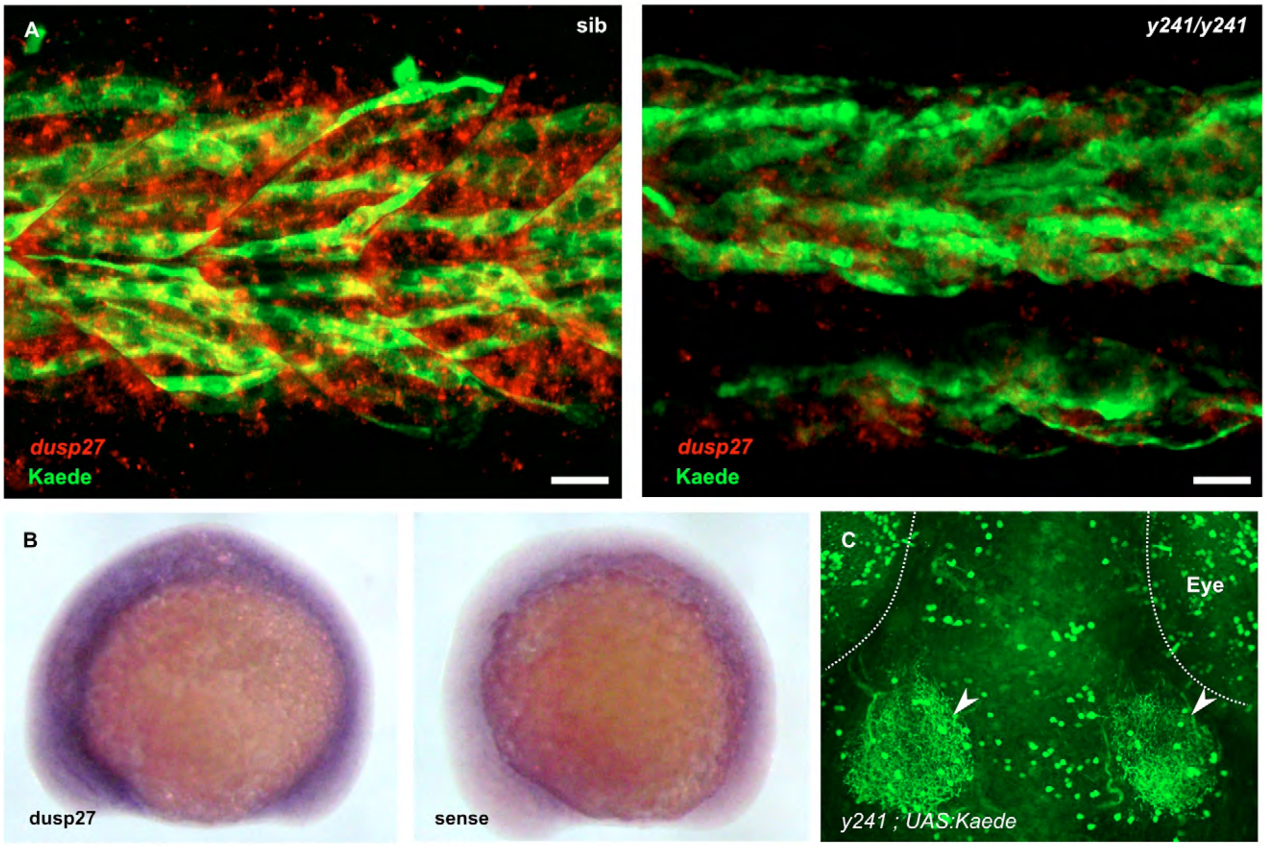

Fig. S2. Additional analysis of *dusp27* expression. (A) Muscle fibers labeled by anti-kaede staining (green) in *y241 ; UAS:Kaede* heterozygotes (left) at 30 hpf co-express *dusp27* (red, *in situ* hybridization), while *dusp27* expression in mutants is strongly diminished. Note that the expression of *dusp27* in fibers not labeled by kaede is likely due to variegated expression of the UAS:Kaede transgene, a commonly observed phenomenon in zebrafish UAS lines (Goll et al., 2009). Scale bar 20 μm. (B) *in situ* hybridization for *dusp27* at 15 somites (right panel shows sense control) (C) In 6 dpf brain, Kaede prominently labels deep tectal neurons that extend neurites into the tectal neuropil (arrowheads) as well as neuronal groups in the retina, cerebellum and hypothalamus (not shown). Scale bar 50 μm.

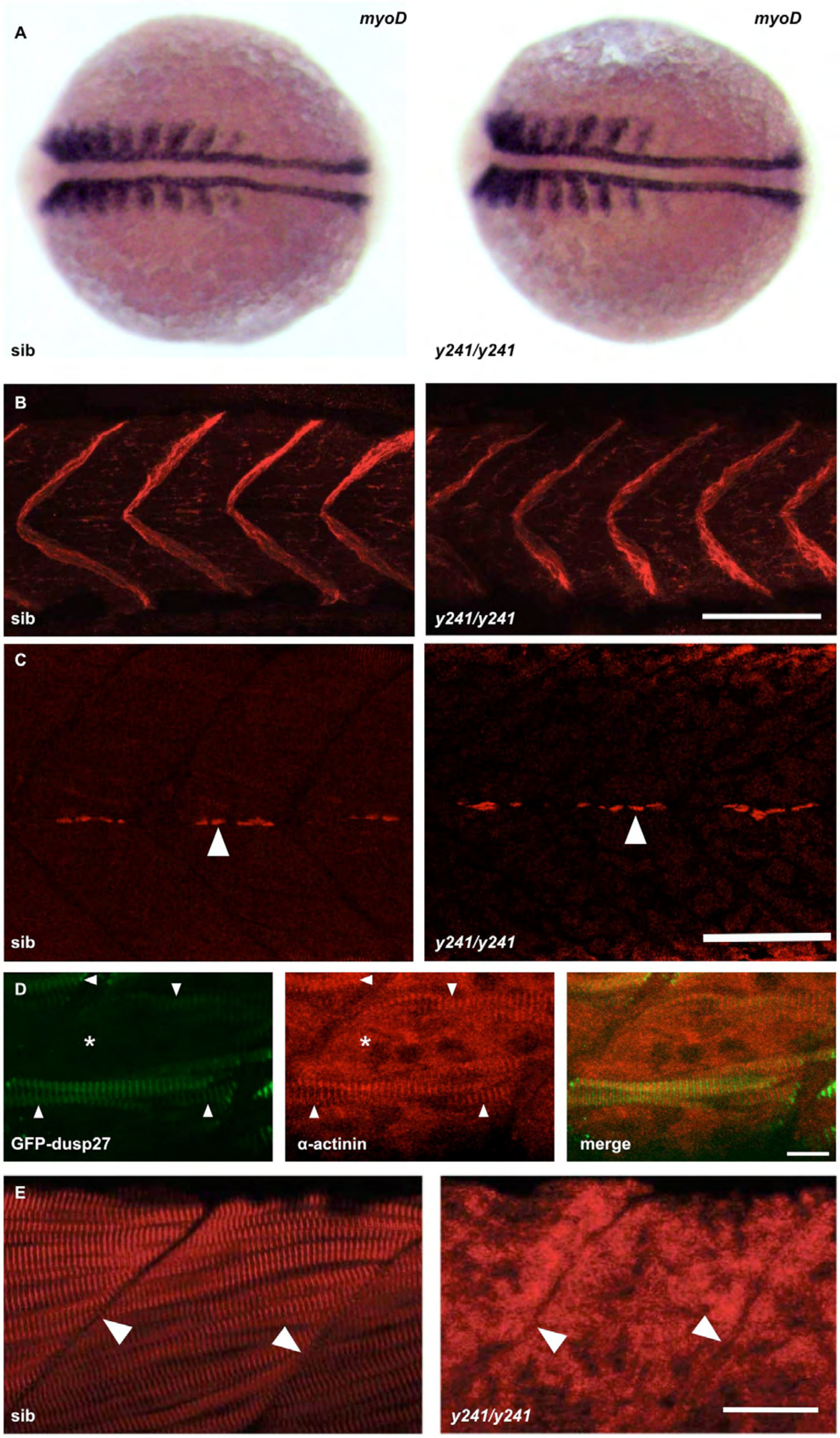

Fig. S3. Additional analysis of muscle markers in *y241* mutants. (A) *In situ* hybridization for *myoD* expression in 10 somite sibling (left) and *y241* mutant (right) embryos. After photographing, embryos were genotyped by PCR. (B) Immunofluorescence staining for dystrophin (merged z-stack through muscle) shows similar localization at somite boundaries in sibling and *y241* mutants in 48 hpf embryos. Scale bar 50 µm. (C) 4D9 staining of engrailed expression in muscle pioneer cells indicates the presence of grossly normal densities of pioneer cells (arrows). Scale bar: 50 um. (D) In *y241* mutants injected with the GFP-dusp27 plasmid, muscle fibers expressing GFP (left panel, arrowheads) recover a normal striated pattern of α-actinin expression (middle panel) while regions of the somite without GFP expression do not show patterned α-actinin expression (asterisk). Merged view on right. Scale bar 10 µm. (E) The normal striated pattern of ryanodine receptor staining is disrupted in 48 hpf *y241* mutant embryos. Arrowheads point to somite boundaries. Scale bar 25 µm.

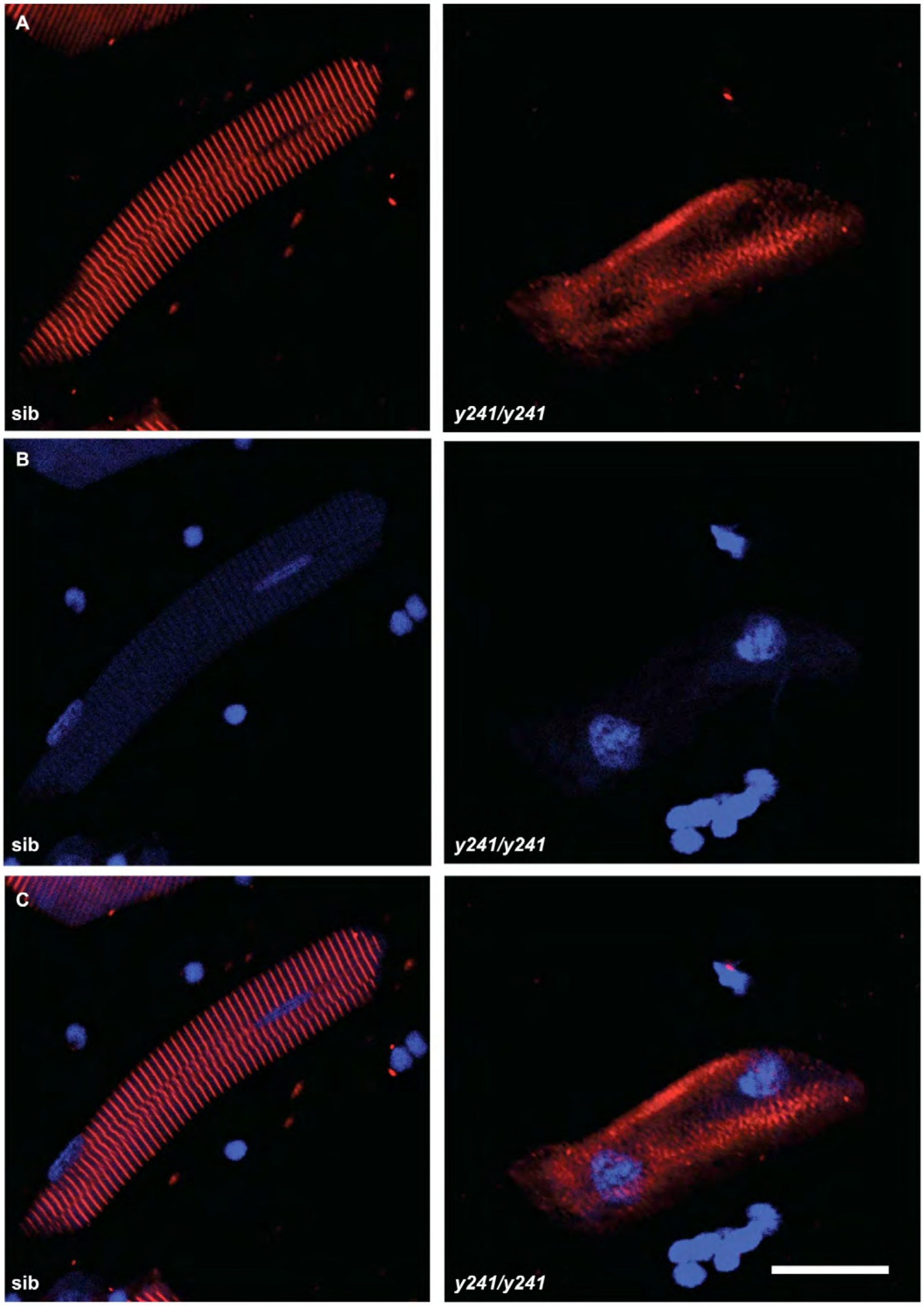

Fig. S4. Immunofluorescence in isolated fast muscle fibers in *y241* mutants. (A) α-actinin stain and (B) DAPI stain in isolated myofibers from 4 dpf sibling and *y241* mutants. Merged image in (C). Scale bar 20 μm.

Supplementary movies. Spontaneous coiling behavior in *dusp27* mutants and siblings (24 hpf).

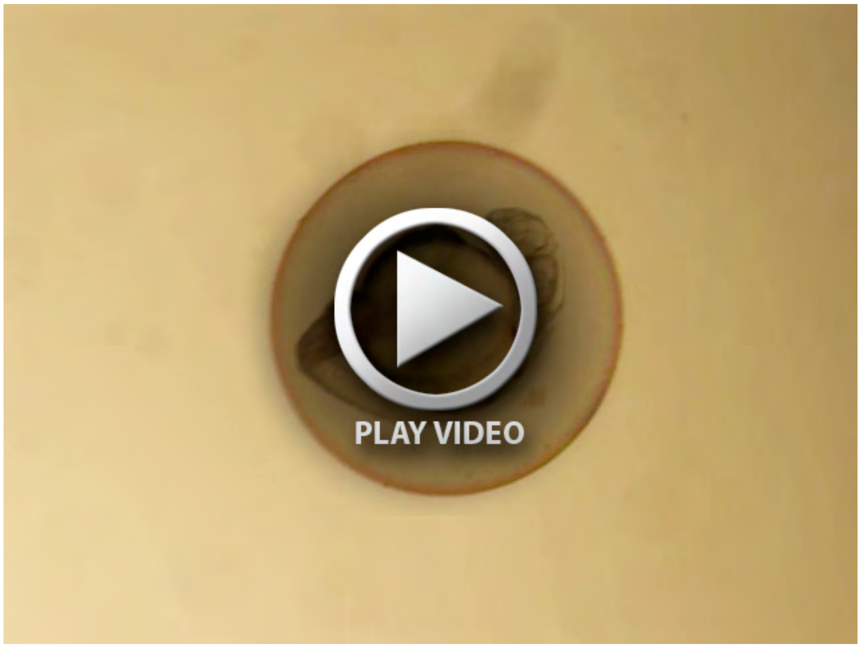

(1) Sibling embryos (23 hpf) show large magnitude tail flexions during spontaneous coiling events.

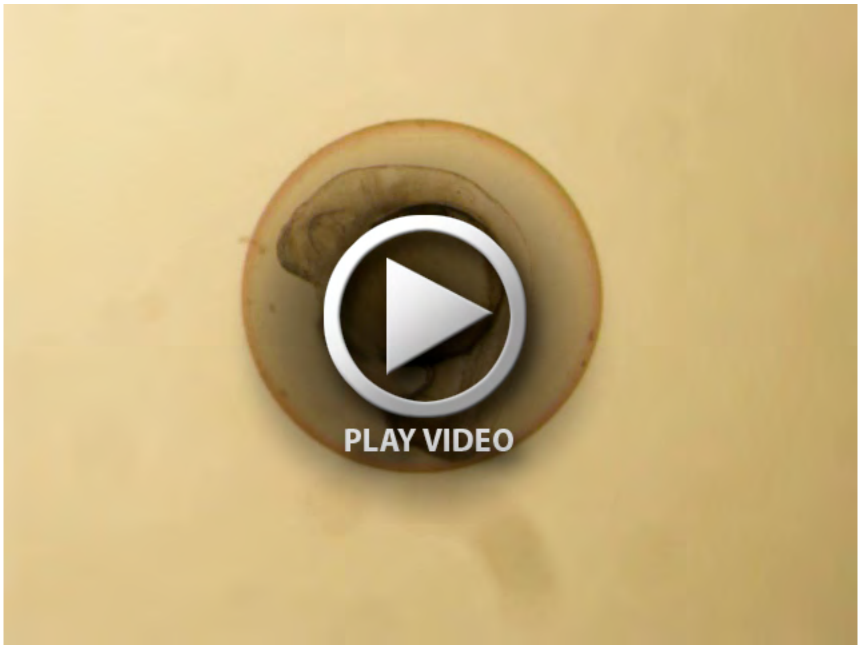

(2) In *y241* mutants (23 hpf), spontaneous coiling events are observed, but with greatly diminished tail flexions.

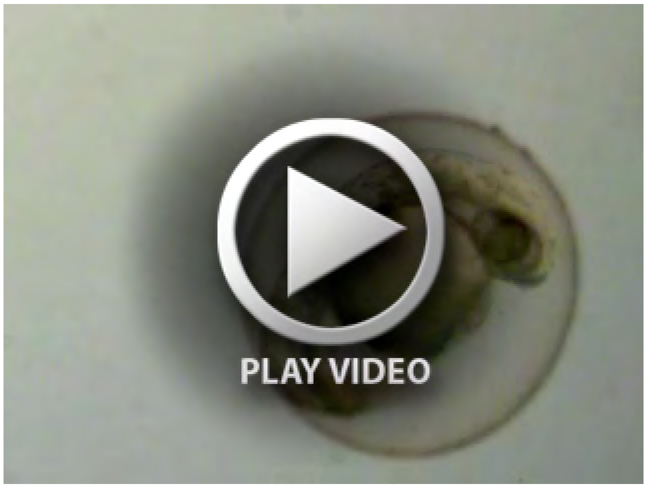

(3) Spontaneous coiling in embryos (28 hpf) injected with control morpholino.

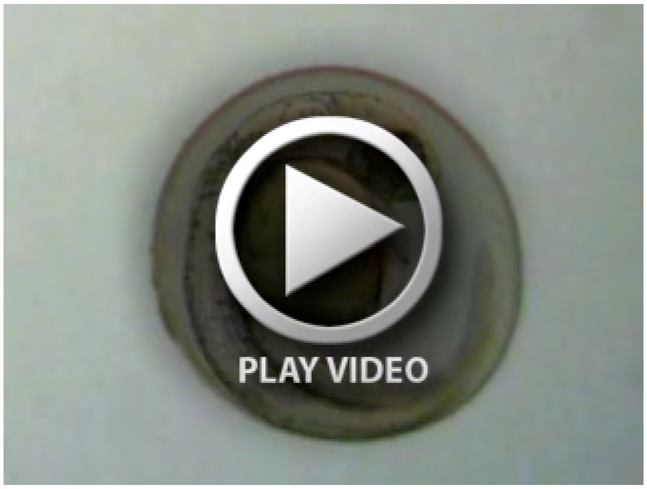

(4) Spontaneous coiling in embryos (28 hpf) injected with morpholino against *dusp27*.
